# The correlates of urinary albumin to creatinine ratio (ACR) in a high risk Australian aboriginal community

**DOI:** 10.1186/1471-2369-14-176

**Published:** 2013-08-16

**Authors:** Zaimin Wang, Wendy E Hoy, Zhiqiang Wang

**Affiliations:** 1 Center for Chronic Disease, School of Medicine, The University of Queensland, Royal Brisbane & Women’s Hospital, Herston, Brisbane, Queensland 4029, Australia

**Keywords:** Albuminuria, Microalbuminuria, Overt albuminuria, ACR, Aboriginal people

## Abstract

**Background:**

Albuminuria marks renal disease and cardiovascular risk. It was estimated to contribute 75% of the risk of all-cause natural death in one Aboriginal group. The urine albumin/creatinine ratio (ACR) is commonly used as an index of albuminuria. This study aims to examine the associations between demographic factors, anthropometric index, blood pressure, lipid-protein measurements and other biomarkers and albuminuria in a cross-sectional study in a high-risk Australian Aboriginal population. The models will be evaluated for albuminuria at or above the microalbuminuria threshold, and at or above the “overt albuminuria” threshold with the potential to distinguish associations they have in common and those that differ.

**Methods:**

This was a cross-sectional study of 598 adults aged 18–76 years. All participants were grouped into quartiles by age. Logistic regression models were used to explore the correlates of ACR categories.

**Results:**

The significant correlates were systolic blood pressure (SBP), C-reactive protein (CRP), uric acid, diabetes, gamma-glutamyl transferase (GGT) (marginally significant, p = 0.054) and serum albumin (negative association) for ACR 17+ (mg/g) for men and 25+ for women. Independent correlates were SBP, uric acid, diabetes, total cholesterol, alanine amino transferase (ALT), Cystatin C and serum albumin (negative association) for overt albuminuria; and SBP, CRP and serum albumin only for microalbuminuria.

**Conclusions:**

This is the most detailed modelling of pathologic albuminuria in this setting to date. The somewhat variable association with risk factors suggests that microalbuminuria and overt albuminuria might reflect different as well as shared phenomena.

## Background

Australian Aboriginal people, especially those living in remote areas, have greater rates of all-cause mortality, cardiovascular death and end-stage renal disease (ESRD) [1-3] compared with other Australian ethnicities. The incidence of patients with ESRD starting renal replacement therapy in remote regions is up to 30 times the national incidence for all Australians [4]. The urine albumin/creatinine ratio (ACR) is commonly used as an index of albuminuria. In a study of one high-risk community, albuminuria, which was pervasive, and much more common than hypertension and diabetes and, not only marked all future risk of renal failure, the treatment of which is very costly, but also predicted cardiovascular and nonrenal noncardiovascular deaths [2,5-8]. Albuminuria was estimated to contribute 75% of the risk of all-cause natural death during a short longitudinal period of observation [1]. Recently the authors reported that albuminuria was still a remarkable predictor for all-cause natural death over an average of 14 years follow-up interval in an Aboriginal community [9]. Thus albuminuria represents a potential target for intervention [10]. A number of variables were measured in a cross-sectional survey on a high-risk Australian Aboriginal population. Thus this study aims to explore the associations between demographic factors, anthropometric index, blood pressure, lipid-protein measurements and other biomarkers and albuminuria. The models will be evaluated for albuminuria at or above the microalbuminuria threshold, and at or above the “overt albuminuria” threshold with the potential to distinguish associations they have in common and those that differ.

## Methods

### Study population

Participants were recruited from an Australian Aboriginal community living in a remote area in Northern Territory of Australia between 1992 and 1998 as part of a screening program for chronic disease. Participants aged 18 years and over were included in the analysis.

### Measurements of baseline characteristics

Urinary albumin concentrations were measured simultaneously by both nephelometric and high-performance liquid chromatography techniques on random urine sample. When we examined over a Z-score continuum of their values, they were identical in defining clinical profiles and predicting deaths [11]. Thus the results here are based on the concentration measured using Beckman immunoassay on a Dade Behring Prospect Analyser, with reagents and calibrators supplied by Dade Behring Diagnostics (Sydney, Australia). Urinary creatinine concentration was measured using an alkaline picrate method (Olympus AU800 Autoanalyzer; interassay CV 2%). Simple anthropometric indices, blood pressure, glucose, lipoprotein profiles, C-reactive protein (CRP), uric acid, serum albumin, cystatin C, serum gamma glutammyl transferase (GGT) and alanine amino transferase (ALT) were also measured. The sex-specific cut points of ACR were applied to categorise participants as, normal (ACR < 17 mg/g for men; <25 for women), microalbuminuria (ACR 17–249 for men; 25–354 for women) and overt albuminuria (ACR ≥ 250 for men; ≥355 for women) [12].

Diabetes refers to those known to be diabetic before the baseline survey or who had fasting glucose ≥126 or 2-hr glucose ≥199.8 mg/dl or “random glucose” ≥199.8 mg/dl. Smoking and alcohol drinking were self-reported. All participants were regrouped into quartiles by age. Continuous variables that are not normal distributed were logarithm-transferred prior to analysis.

### Statistical analysis

Logistic regression modellings for ACR categories including all potential predictors available in this study were applied. All analyses were undertaken using Stata 11.1 (Stata Corp. Stata Statistical Software: Release 11.1, College Station. TX: StatCorp LP, 2009).

The informed consent for the original population screening program was obtained prior to the survey. This study was approved by the Ethics Committee of the Menzies School of Health Research and Territory Health Services and The Behavioural and Social Science Ethical Review Committee of the University of Queensland.

## Results

### Characteristics of participants

At the baseline survey, ACR was measured in a total of 755 participants aged 18 years and over, and 598 of them with a complete set of risk factor measurements were included in the analysis. Age ranged from 18 to 76 years, with a mean of 34 years. The geometric mean, (95% CI) ACR value was 32.6 mg/g (27.5-38.7).

Proportion of participants with normal ACR (using sex-specific cut-off points) was 47.8% while microalbuminuria and overt albuminuria were present in 40.8% and 19.2%, respectively.

BMI, SBP, cholesterol, triglycerides, CRP, cystatin c, serum GGT and diabetes prevalence increased with the age while HDL and serum albumin were opposite (Table 1). Uric acid and serum ALT were not associated with age. The proportion of smokers was significantly high in this community ranged from 67-78% (Table 1).

**Table 1:** Characteristics of participants by age quartile

	**Quartile 1**	**Quartile 2**	**Quartile 3**	**Quartile 4**
	**(n = 150)**	**(n = 151)**	**(n = 149)**	**(n = 148)**
Age (years) (mean, SD)	21.6 (1.8)	27.7 (1.9)	35.8 (2.9)	50.8 (8.2)
BMI (kg/m2) (mean, SD)	21.4 (4.5)	23.6 (4.8)	24.5 (5.4)	24.5 (5.2)
SBP (mmHg) (mean, SD)	116.2 (15.7)	118.8 (15.1)	119.4 (16.7)	130.7 (21.8)
Cholesterol (mg/dL) (mean, SD)	4.12 (0.82)	4.69 (1.03)	4.86 (1.13)	4.99 (1.06)
HDL (mg/dL) (mean, SD)	1.14 (0.26)	1.14 (0.35)	1.12 (0.29)	1.06 (0.22)
Serum albumin (g/l) (mean, SD)	42.1 (4.8)	41.3 (5.7)	39.9 (5.1)	38.5 (4.6)
Uric acid (mg/l) (gmean, 95% CI)	0.34 (0.32–0.36)	0.36 (0.34–0.37)	0.35 (0.34–0.37)	0.35 (0.34–0.36)
Triglyceride (mg/dL) (gmean, 95% CI)	1.20 (1.13–1.28)	1.78 (1.63–1.94)	1.88 (1.70–2.08)	2.08 (1.91–2.25)
CRP (mg/l) (gmean, 95% CI)	3.84 (3.21–4.61)	3.97 (3.35–4.71)	5.74 (4.77–6.89)	6.61 (5.60–7.80)
Cystatin C (mg/l) (gmean,95% CI)	0.67 (0.66–0.69)	0.67 (0.65–0.70)	0.71 (0.68–0.74)	0.76 (0.73–0.80)
Serum ALT (IU/L) (gmean,95% CI)	24.0 (22.4–25.6)	26.7 (24.8–28.9)	23.2 (21.4–25.1)	19.4 (17.9–21.1)
Serum GGT (IU/L) (gmean, 95% CI)	26.9 (24.7–29.2)	36.2 (32.7–40.1)	37.7 (33.7–42.1)	38.0 (34.0–42.5)
Diabetes (%, 95% CI)	4.0 (0.8–7.1)	4.0 (0.8–7.1)	9.4 (4.7–14.1)	28.3 (21.0–35.7)
Smoking (%, 95% CI)	73.3 (66.1–80.5)	78.1 (71.5–84.8)	67.1 (59.5–74.7)	76.3 (69.4–83.2)

### Logistic regression

In the logistic regression modelling, SBP, serum uric acid, CRP and diabetes were significantly associated with albuminuria (Table 2) and it was marginally significant for GGT (p = 0.054) while there was significantly inverse relationship between serum albumin and albuminuria (Table 2). Table 3 shows that the independent correlates of microalbuminuria were SBP, CRP, serum albumin (inverse association) and uric acid (marginally significantly, p = 0.068) while the independent predictors of overt albuminuria were total cholesterol, SBP, uric acid, cystatin c, diabetes, serum albumin and ALT (both were negative association).

**Table 2:** Logistic regression of any albuminuria (ACR ≥ 17 mg/g for men; ≥25 for women)

**Variables**	**Odds ratio**	**95% CI**	**P**
Age	1.15	0.94–1.40	0.181
BMI	0.99	0.95–1.04	0.767
Cholesterol	1.09	0.87–1.37	0.429
HDL	0.63	0.29–1.38	0.253
SBP	1.02	1.01–1.03	**0.004**
Serum albumin	0.91	0.87–0.95	**<0.001**
Serum uric acid*	3.16	1.31–7.61	**0.010**
ALT*	1.01	0.62–1.65	0.995
CRP*	1.29	1.07–1.57	**0.009**
Cystatin C*	1.69	0.66–4.30	0.275
Triglycerides*	1.47	0.93–2.30	0.103
GGT*	1.50	0.98–2.31	**0.054**
Diabetes	2.49	1.18–5.24	**0.016**
Smoking	1.05	0.68–1.64	0.853

*log–transformed.

**Table 3:** Logistic regression modellings for microalbuminuria and overt albuminuria

	**Microalbuminuria**			**Overt albuminuria**		
**Variables**	**Odds ratio**	**95% CI**	**P**	**Odds ratio**	**95% CI**	**P**
Age	1.15	0.94–1.43	0.187	1.11	0.83–1.47	0.491
BMI	0.98	0.94–1.03	0.454	1.04	0.98–1.10	0.183
Cholesterol	0.99	0.78–1.27	0.963	1.49	1.11–1.99	**0.009**
HDL	0.71	0.31–1.62	0.418	0.51	0.15–1.73	0.281
SBP	1.02	1.0–1.03	**0.016**	1.02	1.00–1.03	**0.020**
Serum uric acid	2.42	0.94–6.24	**0.068**	3.66	1.11–12.0	**0.029**
Serum albumin	0.95	0.91–0.99	**0.044**	0.85	0.80–0.90	**<0.001**
ALT*	1.29	0.76–2.20	0.355	0.50	0.27–0.94	**0.031**
CRP*	1.33	1.08–1.64	**0.007**	1.05	0.80–1.38	0.713
Cystatin C*	0.58	0.16–2.06	0.390	8.20	2.68–25.1	**<0.001**
Triglycerides*	1.48	0.92–2.40	0.114	1.48	0.79–2.76	0.225
GGT*	1.33	0.84–2.11	0.202	1.50	0.88–2.58	0.136
Diabetes	1.88	0.83–4.25	0.133	2.41	1.20–4.87	**0.014**
Smoking	1.15	0.71–1.86	0.584	0.74	0.40–1.35	0.326

*log–transformed.

Notes: Microalbuminuria refers to ACR 17–249 mg/g for men and 25–354 for women; Overt albuminuria: ACR ≥ 250 for men and ≥355 for women.

**Figure 1:**
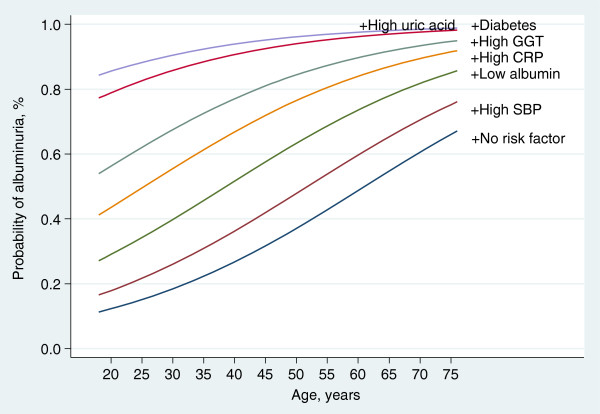
Predicted amplification of albuminuria by multiple risk factors.

**Figure 2:**
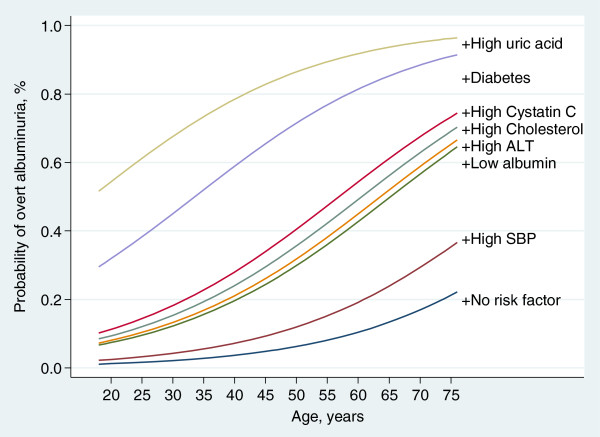
Predicted amplification of overt albuminuria by multiple risk factors.

Figures 1 and 2 shows the predicted amplification of albuminuria and overt albuminuria produced by the simultaneous presence of risk factors identified as independent predictors in the final multivariate model. The predictors were categorical variables derived from each continuous variable using sex-specific median value as the cut-off point. There were substantial rates of elevated ACR (≥17 mg/g for men; ≥25 for women) with increasing age even in the absence of other factors which had independent correlations with ACR. However, the probability of overt albuminuria with increasing age alone was more modest.

## Discussion

In this high risk Aboriginal population, SBP, serum albumin (inverse association), uric acid, CRP, diabetes were significantly associated with albuminuria (p value for GGT was 0.054).

SBP, serum albumin, CRP were significantly associated with microalbuminuria (p value for uric acid was 0.068) while additional risk factors including total cholesterol, ALT, cystatin C and diabetes were significantly associated with overt albuminuria. The shared and the different risk factors for these conditions, traditionally considered to be stages of albuminuria over a continuum, suggest that microalbuminuria and overt albuminuria might reflect different as well as shared phenomena. Different or inconsistent associations of albuminuria with abdominal obesity have been reported in some other studies [13-16] but no significant evidence in this study. Macroalbuminuria was independently associated with hypertension, diabetes and abdominal obesity in Central Australian Aboriginal communities [13].

A close link between high blood pressure and microalbuminuria has been reported by others. In the relationship, higher blood pressure might cause microalbuminuria by increasing glomerular filtration pressure and subsequent renal damage, [17] and adequate control of SBP was associated with low risk for development of microalbuminuria [18]. In addition, the magnitude of urinary albumin excretion determines the severity of BP elevation, [19,20] and there exist heritability and genetic associations between increased urinary albumin excretion and BP in some ethnic groups [21]. CRP, a member of the class of acute phase reactants, is regarded as a sensitive marker of inflammation and was reported as an important contributor to albuminuria (but not an independent predictor for overt albuminuria) in this study. CRP levels are affected significantly by an individual’s genetic makeup and life style [22,23]. People who smoke, have elevated BP, who are overweight or have less physical activity, tend to have higher level of CRP [24,25]. CRP has been reported to be a novel cardiovascular risk factor, [26] and we have reported that it is independently associated with the development of diabetes in this Aboriginal cohort [27]. A significant association between CRP level and microabuminuria has also been reported in some other epidemiological studies [26,28-30]. The relationship might indicate that chronic inflammation is a potential mediator between microalbuminuria and cardiovascular disease (CVD) [28].

A significant inverse association was found between serum albumin and albuminuria, even after adjusting for a number of confounding factors in this study. Albumin is the major protein produced only in the liver. Lower serum albumin may also indicate some conditions other than liver dysfunction. Serum albumin levels are lowered by infection and inflammation, and they decrease as a consequence of acute phase inflammatory reactions, [31] in which increased cytokines switch protein synthesis in the liver from albumin to other phase proteins, and serum albumin falls [32]. Serum albumin levels were also inversely related to age in this study, and, ironically, have been described as higher in smokers than non-smokers [33,34]. Thus, further evaluation in longitudinal studies is required to determine if the inverse relation between serum albumin and albuminuria is causal.

Other reports describe that lower levels of pathologic albuminuria are significantly associated with cigarette smoking and heavy alcohol consumption [35]. However there was no significant association between smoking and albuminuria in our study. Serum GGT has long been used as a liver function test and a marker of excessive alcohol use; [36,37] about 35% of women and 87% of men in this cohort were drinkers, with of men with most males drinking to excess [38]. Associations between high GGT and CVD, [31,32] type-2 diabetes [33] and hypertension [38] have been reported. Our findings support the observation that GGT might also be a novel predictor of chronic kidney disease [39,40]. The possible mechanism of such associations is that oxidative stress depletes glutathione stores which leads to a compensatory increase in GGT, [41,42] or more free radicals might contribute to high levels of GGT [43,44]. There is also evidence that high GGT in certain people is genetically based [45,46]. As serum GGT is easy and cheap to measure, it might have important practical implications on predicting albuminuria and other chronic diseases in Aboriginal communities.

Strengths of this study include the fact that over 80% adult population in this remote Aboriginal community participated. In addition, as Aboriginal people have significantly higher prevalence of chronic kidney disease (CKD), an easy implemented risk prediction model based on routinely obtained laboratory test is important [47]. In this study, all correlates of albuminuria were derived from the available clinical information, and important metabolic markers such as Cystatin C, uric acid and serum albumin were included in the data analysis. Both the albumin and the CRP have helped shed light on the possibly different associations depending on the level of albuminuria. Third, strength is that we now know that these relationships will apply whether ACR is measured by immunoassay rather than HPLC, as we have proven that both are identical in defining clinical profiles and predicting deaths in this community [11].

There were also some limitations in this study. First, the risk predictors in this study were derived from cross-sectional data only, causal association might not exist or the correlates might not be the same in future forecasting. In fact, some correlates are probably markers of the CKD and CVD risk state rather than causal. A 14–16 year longitudinal study in this community is now under analysis and one of reports revealed that albuminuria was still a significant predictor of all-cause natural death after a 14-year interval in this Aboriginal community. However more longitudinal outcomes would shed further light on this issue. Second, with inclusion of new markers, the numbers went down (from 755 down to 598). Those omitted were slightly older (37 yrs vs. 33 yrs), with higher DBP level (78 mmHg vs. 74 mmHg) and higher prevalence of diabetes (16% vs. 6%) than those included. It indicates that this study may underestimate the strength of correlates to ACR in this community. Third, random rather than 24-h urine ACR value was used to define albuminuria in this study. However, the spot ACR is a validated screening test for microalbuminuria and has been used in this setting for 13 years, with widely published data [13]. Finally, findings from a remote community in the Northern Territory of Australia might not be generalizable to other populations. However, most of the significant correlates in our studies have been demonstrated in other settings [7,48-51].

## Conclusions

This is the most detailed modeling of pathologic albuminuria in this setting to date. In this study, SBP, CRP, lower serum albumin, uric acid, GGT and diabetes were significantly associated with albuminuria in the Aboriginal population. The somewhat variable association with risk factors suggests that microalbuminuria and overt albuminuria might reflect different as well as shared phenomena.

## Abbreviations

ACR: Urinary albumin to creatinine ratio; BMI: Body mass index; CKD: Chronic kidney disease; GGT: High gamma-glutamyl transferase; CRP: C- reactive protein (CRP); ESRD: End-stage renal disease; ALT: Alanine amino transferase; HPLC: High-performance Liquid chromatography; SBP: Systolic blood pressure; CVD: Cardiovascular disease.

## Competing interests

All the authors declared no competing interests.

## Authors’ contributions

ZMW - Analysis of data, drafting the article; revising the article, final approval of the version to be published. WH – Design and conduct of field work; Accrual of baselineine and longitudinal data; Conception and interpretation of data; revising the article; providing intellectual content of critical importance to the work described and final approval of the version to be published. ZQW - Revising the article; providing intellectual content of critical importance to the work described and final approval of the version to be published. All authors read and approved the final manuscript.

## Authors’ information

ZMW - Ph.D, research fellow/epidemiologist at Centre for Chronic Disease, School of Medicine, The University of Queensland, Australia.

W H - FRACP, Professor, Director at Centre for Chronic Disease, School of Medicine, The University of Queensland, Australia.

ZQW – PhD, Associate Professor, deputy Director at Centre for Chronic Disease, School of Medicine, The University of Queensland, Australia.

## Pre-publication history

The pre-publication history for this paper can be accessed here:

http://www.biomedcentral.com/1471-2369/14/176/prepub

## References

[B1] Spencer J Silva D Hoy W An epidemic of renal failure among Australian aboriginal Med J Aust 1998 168 537 541 9640302 10.5694/j.1326-5377.1998.tb139080.x

[B2] Cunningham J Condon JR Premature mortality in aboriginal adults in the Northern Territory Med J Aust 1996 165 309 312 8862329 10.5694/j.1326-5377.1996.tb124987.x

[B3] Veroni M Gracey M Rouse I Patterns of mortality in Western Australian aboriginals, 1983–89 Int J Epidemiol 1994 23 73 81 10.1093/ije/23.1.73 8194927

[B4] Cass A Cunningham J Wang Z Hoy W Regional variation in the incidence of end-stage renal disease in Indigenous Australians Med J Aust 2001 175 24 27 11476198 10.5694/j.1326-5377.2001.tb143507.x

[B5] Wirta O Pasternack A Mustonen J Laippala P Renal and cardiovascular predictors of 9-year total and sudden cardiac mortality in non-insulin-independent diabetic subjects Nephrol Dial Transplant 1997 12 2612 2617 10.1093/ndt/12.12.2612 9430860

[B6] Hoy WE Wang Z Baker PRA Kelly AM Reduction in natural death and renal failure from a systematic screening and treatment program in an Australian aboriginal community Kidney Int 2003 63 Suppl 83 S66 S73 10.1046/j.1523-1755.63.s83.14.x 12864878

[B7] Hoy W McDonald SP Albuminuria: marker or target in Indigenous populations Kidney Int 2004 66 Suppl 92 S25 S31 10.1111/j.1523-1755.2004.09207.x 15485412

[B8] Hoy WE Renal disease in aboriginal Australians Med J Aust 1996 165 126 127 8709872 10.5694/j.1326-5377.1996.tb124882.x

[B9] Wang Z Hoy WE The predictive value of albuminuria for renal and nonrenal natural deaths over 14 years follow-up in a remote aboriginal community Clin Kidney J 2012 5 519 525 10.1093/ckj/sfs125 PMC4400551 26064480

[B10] McDonald SP Wang Z Hoy WE Physical and biochemical predictors of death in an Australian aboriginal cohort Clin Expl Pharmacol P 1999 26 618 621 10.1046/j.1440-1681.1999.03104.x 10474776

[B11] Wang Z Hoy W Nicol JL Su Q Atkins R Polkinghome KR Predictive value of nephelometric and high-performance liquid chromatography assays of urine albumin for mortality in a high –risk aboriginal population Am J Kidney Dis 2008 52 672 682 10.1053/j.ajkd.2008.03.007 18585832

[B12] Warram JH Gearin G Laffel L Krolewski AS Effect of duration of type I diabetes on the prevalence of stages of diabetic nephropathy defined by urinary albumin/creatinine ratio J Am Soc Nephrol 1996 7 930 937 8793803 10.1681/ASN.V76930

[B13] Rowley KG Iser DM Best J O’Dea K Leonard D McDermitt R Albuminuria in Australian aboriginal people: prevalence and associations with components of the metabolic syndrome Diabetologia 2000 43 1397 1403 10.1007/s001250051545 11126409

[B14] Jager A Kostense PJ Nijpels G Heine RJ Bouter LM Stehouwer CDA Microalbuminuria is strongly associated with NIDDM and hypertension, but not with the insulin resistance syndrome: the Hoorn study Diabetologia 1998 41 694 700 10.1007/s001250050970 9662052

[B15] Goetz FC Jacobs DR Chavers B Roel J Yelle M Sprafka JM Risk factors for kidney damage in the adult population of Wadena, Minnesota Am J Epidemiol 1997 145 91 102 10.1093/oxfordjournals.aje.a009091 9006305

[B16] Smulders YM Rakic M Stehouwer CDA Weijers RN Slaats EH Silberbusch J Determinants of progression of microalbuminuria in patients with NIDDM Diabetes Care 1997 20 999 1005 10.2337/diacare.20.6.999 9167114

[B17] Koroshi A Microalbuminuria, is it so important? Hippokratia 2007 11 105 107 19582202 PMC2658722

[B18] Glassock RJ Prevention of microalbuminuria in type-2 diabetes: millimetres or milligrams J am Soc Nephrol 2006 17 3276 3278 10.1681/ASN.2006101131 17108313

[B19] Hodge AM Dowse GK Zimmet PZ Microalbuminuria, cardiovascular risk factors, and insulin resistance in two populations with a high risk of type 2 diabetes mellitus Diabet Med 1996 13 441 449 10.1002/(SICI)1096-9136(199605)13:5<441::AID-DIA99>3.0.CO;2-J 8737026

[B20] Konen JC Summerson JH Bell RA Curtis LG Racial differences in symptoms and complications in adults with type-2 diabetes mellitus Ethn Health 1999 4 39 49 10.1080/13557859998182 10887461

[B21] Guo X Cui J Wagenknecht LE Norris JM Haffner SM Darwin C Jinagouda S Rotter JI Saad MF Cosegragation of albuminuria and blood pressure: the InsulinResistance Atherosclerosis (IRAS) family study Am J Hypertens 2005 18 823 827 10.1016/j.amjhyper.2005.01.022 15925742

[B22] Li SH Szmitko PE Weisel RD Wang CH Fedak PWM Li RK Mickle DAG Verma S C-reactive protein upregulates complement-inhibitory factors in endothelial cells Circulation 2004 109 833 836 10.1161/01.CIR.0000117087.27524.0E 14967730

[B23] Albert MA Glynn RJ Buring J Ridker PM C-reactive protein levels among women of various ethnic groups living in the United Status (from the Women’s Health Study) Am J Cardiol 2004 93 1238 1242 10.1016/j.amjcard.2004.01.067 15135696

[B24] Greenfield JR Samaras K Jenkins AB Kelly PJ Spector TD Gallimore JR Pepys MB Campbell LV Obesity is an important determinant of baseline serum C-reactive protein concentration in monozygotic twins, independent of genetic influences Circulation 2004 109 3022 3028 10.1161/01.CIR.0000130640.77501.79 15184288

[B25] Chien KL Hsu HC Chen MF Lee YT Association of C-reactive protein, smoking and metabolic syndrome among the health check-up population Acta Cardio Sin 2005 21 98 104

[B26] Nakamura M Onoda T Itai K Ohsawa M Satou K Sakai T Segawa T Sasaki J Tonari Y Hiramori K Okayama A Association between serum c-reactive protein levels and microalbuminuria: a population-based cross-sectional study in northern Iwate, Japan Internal Med 2004 43 919 925 10.2169/internalmedicine.43.919 15575240

[B27] Wang Z Hoy WE C- reactive protein and the risk of developing type 2 diabetes in aboriginal Australians Diabetes Res Clin Pr 2007 76 37 43 10.1016/j.diabres.2006.07.018 16952410

[B28] Festa A D’agostino R Howard G Mykkanen L Tracy RP Haffner SM Inflammation and microalbuminuria in nondiabetic and type 2 diabetic subjects: the insulin resistance atherosclerosis study Kidney Int 2000 58 1703 1710 10.1046/j.1523-1755.2000.00331.x 11012904

[B29] Stuveling EM Bakker SJL Hillege HL Burgeruhof JGM Jone PED Gans ROB De ZD C-reactive protein modifies the relationship between blood pressure and microalbuminuria Hypertension 2004 43 791 796 10.1161/01.HYP.0000120125.08867.42 14967837

[B30] Tsioufis C Dimitriadis K Chatzis D Vasiliadou C Tousoulis D Papademstriou V Toutouzas P Stefanadis C Kallikazaros I Relation of microalbuminuria to adiponectin and augmented C-reactive protein levels in men with essential hypertension Am J Cardio 2005 96 946 951 10.1016/j.amjcard.2005.05.052 16188522

[B31] Jousilahti P Rastenyte D Tuomilehto J Serum gamma-glutamyl transferase, self-reported alcohol drinking and the risk of stroke Stroke 2000 31 1851 1855 10.1161/01.STR.31.8.1851 10926946

[B32] Lee DH Silventoinen K Hu G Jacobs DR Jr Jousilahti P Sundvall J Tuomilehto J Serum gamma-glutamyltransferase predicts non-fatal myocardial infarction and fatal coronary heart disease among 28, 838 middle-aged men and women Eur Heart J 2006 27 2170 2176 10.1093/eurheartj/ehl086 16772340

[B33] Meisinger C Lowel H Heier M Schneider A Thorand B Serum gamma- glutamyltransferase and risk of type 2 diabetes mellitus in men and women from the general population J Intern Med 2005 258 527 535 10.1111/j.1365-2796.2005.01572.x 16313476

[B34] Lee DH Jacobs DR Jr Gross M Kiefe CI Roseman J Lewis CE Steffes M Gamma-glutamyltransferase is a predictor of incident diabetes and hypertension: the coronary artery risk development in young adults (CARDIA) study Clin Chem 2003 49 1358 1366 10.1373/49.8.1358 12881453

[B35] Lee DH Jacobs DR Jr Gross M Steffes M Serum gamma-glutamyltransferase was differently associated with microalbuminuria by status of hypertension or diabetes: the coronary artery risk sevelopment in young adults (CARDIA) study Clin Chem 2005 51 1185 1191 10.1373/clinchem.2004.045872 15890893

[B36] Hoy WE Wang Z VanBuynder P Backer PRA McDonald SM Mathews JD The natural history of renal disease in Australian aborigines: part 2 albuminuria predicts natural death and renal failure Kidney Int 2001 60 249 256 10.1046/j.1523-1755.2001.00793.x 11422758

[B37] Conlgrave KM Saunders JB Reznik RB Whitfield JB Prediction of alcohol-related ham by laboratory test results Clin Chem 1993 39 2266 2270 7900934

[B38] Hoy WE Mathews JD McCredie DA Pugsley DJ Havhurst BG Rees M Kile E Walker KA Wang Z The multidimensional nature of renal disease: rates and associations of albuminuria in an Australian aboriginal community Kidney Int 1998 54 1296 1304 10.1046/j.1523-1755.1998.00099.x 9767547

[B39] Conigrave KM Degenhardt LJ Whitefield JB Saunders JB Helander A Tabakoff B CDT, GGT, and AST as markers of alcohol use: the WHO/ISBRA collaborative project Alcohol Clin Exp Res 2002 26 332 339 10.1111/j.1530-0277.2002.tb02542.x 11923585

[B40] Whitfield JB Serum -glutamyltransferase and risk of disease Clin Chem 2007 1 1 2 17202494 10.1373/clinchem.2006.080911

[B41] Whitfield JB Gamma glutamyl transferase Crit Rev Clin Lab Sci 2001 38 263 355 10.1080/20014091084227 11563810

[B42] Lee DH Blomhoff R Jacobs DR Jr Is serum gamma glutamyltransferase a marker of oxidative stress? Free Radic Res 2004 38 535 539 10.1080/10715760410001694026 15346644

[B43] Paolicchi A Tongiani R Tonarelli P Comporti M Pompella A Gamma- glutamyl transpeptidase-dependent lipid peroxidation in isolated hepatocytes and HepG2 hepatoma cells Free Radic Biol Med 1997 22 853 860 10.1016/S0891-5849(96)00422-4 9119254

[B44] Drozdz R Parmentier C Hachad H Leroy P Siest G Wellman M Glutamyltransferase dependent generation of reactive oxygen species from a glutathione/transferrin system Free Radic Biol Med 1998 25 786 792 10.1016/S0891-5849(98)00127-0 9823544

[B45] Bathum L Petersen HC Rosholm JU Hyltoft PP Vaupel J Christensen K Evidence for a substantial genetic influence on biochemical liver function tests: results from a population-based Danish twin study Clin Chem 2001 47 81 87 11148181

[B46] Whitfield JB Zhu G Nestler JE Heath AC Martin NG Genetic covariation between serum gamma-glutamyltransferase activity and cardiovascular risk factors Clin Chem 2002 48 1426 1431 12194918

[B47] Tangri N Stevens LA Griffith J Tighiouart H Djurdjev O Naimark D Levin A Levey AS A predictive model for progression of chronic kidney disease to kidney failure JAMA 2011 305 1553 1559 10.1001/jama.2011.451 21482743

[B48] Huo L Xu M Li R Dai M Wang JG Ning G Li XY Prevalence and prevalence of microalbuminuria in persons with various glucose tolerance levels ZhongHua YiXue Za Zhi 2007 87 2537 2540 18067826

[B49] Anvari MS Boroumand MA Pourgholi L Sheikhfathollahi M Rouhzendeh M Rabbani S Goodarzynejad H Potential link of microalbuminuria with metabolic syndrome in patients undergoing coronary angiography Arch Med Res 2009 40 399 405 10.1016/j.arcmed.2009.06.011 19766905

[B50] Agrawal V Vanhecke TE Rai B Franklin BA Sangal RB McCullough PA Albuminuria and renal function in obese adults evaluated for obstructive sleep apnea Nephron Clin Prac 2009 113 c140 c147 10.1159/000232594 19672111

[B51] Prasad GV Bandukwala F Huang M Zaltman JS Microalbuminuria post-renal transplantation: relation to cardiovascular risk factors and C-reactive protein Clin Transplant 2009 23 313 320 10.1111/j.1399-0012.2008.00913.x 19537299

